# Editorial: Advances in Tribochemistry

**DOI:** 10.3389/fchem.2022.925015

**Published:** 2022-06-06

**Authors:** Lei Chen, Andreas Rosenkranz, Lev S. Rapoport, Seong H. Kim

**Affiliations:** ^1^ State Key Laboratory of Traction Power, School of Mechanical Engineering, Tribology Research Institute, Southwest Jiaotong University, Chengdu, China; ^2^ Department for Chemical Engineering, Biotechnology and Materials, University of Chile, Santiago, Chile; ^3^ Holon Institute of Technology, Holon, Israel; ^4^ Department of Chemical Engineering and Materials Research Institute, The Pennsylvania State University, University Park, PA, United States

**Keywords:** tribochemistry, tribochemical wear, adhesive interaction, triboelectrochemical reaction, superlubricity, interfacial bonding

Shear-induced mechanochemical reactions (or so-called tribochemical reactions) occur ubiquitously in sliding interfaces, which subsequently alter friction and wear of the interface. Such reactions play an essential role in all length scales from rock friction in earthquakes (Li, et al., 2011), to interfacial lubrication of macro-scale mechanical components (Liu, et al., 2021), and even the adhesive wear of micro/nano-electromechanical systems (Chen and Qian, 2021). Recent technological advances (such as atomic force microscopy, transmission electron microscopy and other microscopic approaches) together with numerical simulations (such as reactive molecular dynamics simulations and density functional theory calculations) offer promising approaches to elucidate the molecular details and driving forces for tribo-chemical reactions. In this Research Topic, seven contributions including two reviews and five original research articles on the latest advances in tribochemistry are compiled.

First, Liu et al. studied the dynamic evolution of friction and surface morphology on Cu plates caused by triboelectrochemical reactions in pure water and pure base oils under external electric stimulation. This article proposed a chemical potential equation based on the effective collision model of chemical reactions to explain how the electrical and mechanical contributions affect the observed triboelectrochemical phenomena in experiments.

Gao et al. report the macroscale superlubricity phenomenon at a temperature of 300 ° C for surfaces produced by burnishing powders of antimony trioxide (Sb_2_O_3_) and magnesium silicate hydroxide coated with carbon (MSH/C) onto nickel superalloy substrates. *In-situ* chemical analysis together with other atomic structure characterization indicated that the superlubricity nature of the newly designed MSH/C-based coating could be attributed to the synergistic effect of antimony oxide adhesion layer, tribochemically-active MSH powders, and a tribochemically-generated superficial carbon film.

Tan et al. verified microscale superlubricity for the uniform and ordered self-assembly of several liquid crystals on a highly-oriented pyrolytic graphite surface. Combined with density functional theory calculations, they confirmed the positive correlation between friction and interfacial interaction strength, thus suggesting the dominant contribution for energy dissipation to be the continuous formation, breaking and reformation of physical bonds.

To understand the interfacial lubrication mechanism in detail, Zuo et al. conducted molecular dynamics simulations to reveal the adhesive interaction between two rubbing surfaces made of Fe and polytetrafluoroethylene (PTFE). The interfacial adhesion was investigated as a function of the surface orientation of Fe and the chemical functionality of PTFE molecules. They clarified that the adhesion interaction between Fe and the adsorbed PTFE transfer film can be attributed to the van der Waals force originating from the iron atoms of the Fe surface and the F atoms of the adsorbate film.

Mechanochemical reaction may not only determine the lubrication properties, but also play an important role in ultra-precision surface manufacturing. Considering chemical mechanical polishing (CMP), the planarization process can be viewed as the controllable atomic material removal by adjusting tribochemical reactions. Guo et al. compared the mechanochemical wear of oxide-free and oxidized GaN surfaces rubbed against Al_2_O_3_ nano-asperities as a function of ambient humidity. The tribochemical reactions occurring in that interface were described by mechanically-assisted Arrhenius-type kinetics model. This further indicated that the outermost surface oxide layer enlarges the energy barrier for the initiation of the mechanochemical atomic attrition, resulting in low nanoscale wear of oxidized GaN compared to oxide-free specimen.

Michalchuk et al. reviewed the overall Research Topic “tribochemistry” (or named mechanochemistry, mechanical alloying). Moreover, they discuss the experimental parameters or conditions that are indispensable when describing tribochemical reactions, which is helpful for checking the reproducibility of experimental data, whilst also making comparison possible, particularly when different experimental conditions have been employed. The main type of mechanical interaction and the critical parameters determining the activation of the tribochemical reactions are encoded in a clear, concise, and self-explanatory way.

Finally, Luo et al. review the latest developments in the tribochemical wear of 2D materials (graphene, h-BN, MoS_2_), carbon bulk materials (diamond, DLC films), silicon-based materials (silicon and silicon oxide, silicon-based ceramics and silicate glasses) and metals (Al and Cu), which are commonly used as solid lubricants, tribo-elements, or structural materials in micro/nano-electromechanical devices. Based on theoretical and experimental results, the underlying tribochemical wear mechanisms and processes are discussed, with a particular focus on the formation of interfacial bonds.

As guest editors, we hope that this Research Topic covering the latest advancements of tribochemistry can serve as a useful guide for researchers and engineers in similar or related fields. The collection contributes to the field due to the universality of its focus on mechanically stressed, dynamic contacts. We thank all contributors (authors and co-authors) for their excellent work and all reviewers for their selfless dedication. We also thank the editorial staff of Frontiers in Chemistry for providing valuable assistance in the entire editing process.
